# Visual attention to dynamic faces and objects is linked to face processing skills: a combined study of children with autism and controls

**DOI:** 10.3389/fpsyg.2013.00185

**Published:** 2013-04-10

**Authors:** Julia Parish-Morris, Coralie Chevallier, Natasha Tonge, Janelle Letzen, Juhi Pandey, Robert T. Schultz

**Affiliations:** ^1^Departments of Pediatrics and Psychiatry, Perelman School of Medicine, University of PennsylvaniaPhiladelphia, PA, USA; ^2^Center for Autism Research, Children's Hospital of PhiladelphiaPhiladelphia, PA, USA; ^3^Department of Clinical and Health Psychology, University of FloridaGainesville, FL, USA

**Keywords:** autism, eye tracking, face processing, eyetracking, autism spectrum disorder, ASD

## Abstract

Although the extant literature on face recognition skills in Autism Spectrum Disorder (ASD) shows clear impairments compared to typically developing controls (TDC) at the group level, the distribution of scores within ASD is broad. In the present research, we take a dimensional approach and explore how differences in social attention during an eye tracking experiment correlate with face recognition skills across ASD and TDC. Emotional discrimination and person identity perception face processing skills were assessed using the Let's Face It! Skills Battery in 110 children with and without ASD. Social attention was assessed using infrared eye gaze tracking during passive viewing of movies of facial expressions and objects displayed together on a computer screen. Face processing skills were significantly correlated with measures of attention to faces and with social skills as measured by the Social Communication Questionnaire (SCQ). Consistent with prior research, children with ASD scored significantly lower on face processing skills tests but, unexpectedly, group differences in amount of attention to faces (vs. objects) were not found. We discuss possible methodological contributions to this null finding. We also highlight the importance of a dimensional approach for understanding the developmental origins of reduced face perception skills, and emphasize the need for longitudinal research to truly understand how social motivation and social attention influence the development of social perceptual skills.

## Introduction

Face recognition is one of the more thoroughly studied skills in the field of autism research (Wolf et al., 2008; Tanaka et al., 2012; for a reviews see Harms et al., 2010; Weigelt et al., 2012). While some aspects of typical face recognition may be preserved among individuals with autism spectrum disorder (ASD; for example, aspects of holistic processing: Scherf et al., 2008; Faja et al., 2009), research on face identity recognition and facial expression recognition consistently reveal impairments relative to typically developing children (TDC; Wolf et al., 2008; McPartland et al., 2011; Tanaka et al., 2012).

Face processing is believed to be a universal domain of expertise in humans and perhaps one of the earliest to develop (Gliga and Csibra, 2007). Early functional specialization for faces during infancy contrasts with that of other categories of objects, such as body parts (Gliga and Csibra, 2007) and may result from special attention to social information throughout development, allowing for more perceptual discrimination and categorization experience with face stimuli. Evidence for this early attentional bias is robust. Classic studies have demonstrated that despite poor vision, newborns display a preference for looking at face-like stimuli within days or even hours after birth (Goren et al., 1975; Johnson and Morton, 1991) and recent research has highlighted that this attentional bias bears the signature of a domain-specific disposition to preferentially process faces (Rosa-Salva et al., 2010). Even if this bias is present from early in life in our species, individual differences in the prioritization of social information by attention and perceptual systems may yield individual differences in measured social perceptual skills later in childhood, thereby creating a continuum of skill within the population (Schultz, 2005; Russell et al., 2009).

Reduced attention to and motivation for engaging with face stimuli is a prominent hypothesis for why children with ASD might, on average, have reduced face perceptual skills (Schultz et al., 2000; Grelotti et al., 2002; Dawson et al., 2005; Chevallier et al., 2012a,b). According to this social motivation hypothesis, faces have a significantly less reward value for most children with ASD, leading to reduced social attention and diminished social experience which blunts the development of cortical specialization for faces (Grelotti et al., 2002; Johnson, 2005; Schultz, 2005). Reduced motivation is thus seen as ultimately depriving children with ASD of the visual experience needed to develop their face perception skills. This hypothesis is consistent with infrared gaze tracking studies showing that individuals with autism attend more to the non-social than social features of static visual scenes (Riby and Hancock, 2008; Sasson et al., 2008). Similarly, in studies using dynamic movie clips, children, adolescents and young adults with autism fixate less on people, faces and eyes and more on objects than do typical controls (Klin et al., 2002; Nakano et al., 2010; Rice et al., 2012).

Differences in social attention appear to be one of the earliest signs of autism. For example, a preference for non-social patterns (e.g., geometric shapes) in toddlers is a robust risk factor for developing the disorder (Pierce et al., 2011), and differential electrophysiological responses to shifts in eye gaze at 6 months predict ASD group membership nearly 3 years later (Elsabbagh et al., 2012).

The present research aims to provide a more direct test of the link between social attention and face perception by examining spontaneous attention to faces and objects in participants occupying the entire face expertise continuum. Prior research using ASD and typical participants has focused on group means, overlooking within-group variability. An alternative approach is to ignore diagnostic categories and boundaries and adopt a more dimensional approach (Insel et al., 2010; Sanislow et al., 2010). Dimensional approaches are especially promising in the context of developmental models that propose links between observable behaviors and developmental outcomes.

In the present study, participants' gaze was tracked as they watched movies of actors showing different facial expressions and videos of non-social moving objects (e.g., a bulldozer pushing earth, clothes on a line flapping in the wind) in the same display. The four videos composed a 2 by 2 design, faces vs. objects that were either of high vs. low salience (e.g., faces gazing directly at the camera vs. averted; bulldozers vs. clothing). This study tested the following hypotheses:

1. Attention to faces correlates with face perception accuracy as measured by two subtests of the *Let's Face It!* Skills Battery across all participants.
2. Social skills (as measured by the SCQ) predict social attention and face perception skill.
3. On average, the ASD group will score lower on face perception tests and will spend less time attending to social information.

## Methods

### Participants

We studied 110 children and adolescents, including 60 diagnosed with an ASD (7 female) and 50 typically developing controls (TDC; 12 female). ASD and TDC groups were matched on non-verbal cognitive ability as measured by the Differential Ability Scales, Second Edition (DAS-II, Elliot, 2007), gender ratio and chronological age (Table 1). Participants had no uncorrected auditory or visual impairment, known genetic conditions, history of TBI, premature birth, or other medical or neurological abnormality. All participants were native speakers of English. Members of the TDC group did not have a DSM-IV-TR Axis I disorder. Data validity across the eyetracking portion of the experiment was examined and inclusionary criterion required participant recordings to have a sampling rate above 80% (as calculated by the Tobii software). Initial screening for autism symptomatology was conducted using the Social Communication Questionnaire (SCQ; Rutter et al., 2003a,b) and severity of symptom presentation was documented using the Social Responsiveness Scale (SRS; Constantino and Gruber, 2005). SCQ lifetime scores were also used to test correlational hypotheses. Current diagnosis was confirmed by expert clinical judgment, based on parent-reported developmental history (Autism Diagnostic Interview-Revised: ADI-R; Rutter et al., 2003a,b) and symptom presentation (Autism Diagnostic Observation Schedule: ADOS; Lord et al., 2000). Within the ASD cohort, 52 children were given the ADOS module 3 and eight children were given module 4. Using the original ADOS algorithm (Lord et al., 2000), 31 scored in the autism range, 22 scored in the ASD range, and 7 children scored below ADOS diagnostic cutoffs (see Table 2), but nevertheless met criteria according to developmental history and expert clinical judgment (Lord et al., 2000). Total scores were also tabulated using the revised ADOS algorithm (Gotham et al., 2009) for those individuals who received module 3 of the ADOS (currently, no revised algorithm exists for module 4). Based on the revised algorithm, 37 participants scored within the autism range, 7 scored within the ASD range, and 8 scored below cutoffs. Each revised algorithm score was converted to a standardized autism symptom severity score, following the procedures described by Gotham and colleagues (2009). When using this symptom severity metric, 8 participants were classified as non-spectrum, 6 ASD, and 38 AUT (autism). All assessment measures were administered, scored, and interpreted by a clinical psychologist or supervised doctoral level psychology trainee who met standard requirements for research reliability.

**Table 1:** **Participant characteristics by diagnostic group**.

	**ASD (*N* = 60)**	**TDC (*N* = 50)**	***t*-value**	***p*-value**
Mean age in years (SD)	11.28 (2.89)	11.34 (3.04)	0.10	0.92
Age range	6.17–17.92	6.33–17.92		
Mean GCA (SD)	111.63 (14.61)	113.70 (14.58)	0.74	0.46
GCA range	88–158	87–150		
Mean verbal (SD)	110.12 (16.61)	116.42 (16.70)	1.98	0.05
Verbal score range	77–161	89–165		
Mean non-verbal (SD)	111.07 (15.48)	108.26 (13.71)	−1.00	0.32
Non-verbal range	84–166	80–143		
Mean LFI score (SD)	78.83 (7.29)	82.70 (7.78)	2.69	0.008
LFI range	61.67–96.66	65.00–96.66		
Mean SCQ score (SD)	20.67 (5.61)	1.12 (1.29)	−24.11	0.000
SCQ range	11–34	0–4		
			Chi-Square	*p*-value
Sex: Male	53 of 60	38 of 50	2.90	0.09

**Table 2:** **Mean ADOS scores (original algorithm)**.

	**Communication mean (SD)**	**Social interaction mean (SD)**	**Total mean (SD)**
Module 3 (*N* = 52)	2.94 (1.29)	7.08 (2.73)	10.02 (3.69)
Module 4 (*N* = 8)	3.57 (1.72)	7.13 (1.46)	10.50 (2.83)

A communication score of *2* indicates ASD, and *3* or above indicates autism. A social interaction score of *4* or *5* indicates ASD, and *6* or above indicates autism. Total scores of *10* or above indicate autism; total scores of *7* or above indicate ASD.

### Measures and design

The *Let's Face It!* Skills Battery (LFI; Wolf et al., 2008; Tanaka et al., 2012) is composed of 11 separate computer-administered tests, guided by contemporary theories of face perception processes. It assesses face recognition abilities in two broad domains involving (1) the perception of person identity and (2) the perception of facial expression. These constructs have been validated in other samples using principal components analyses (Wolf et al., 2008). Previously, Wolf and colleagues (2008) and Tanaka and colleagues (2012) found robust deficits (standardized effect sizes ranging from 0.40 to 1.0 SD) in both person and emotion identity using a common large sample (~66–85 individuals with ASD and 66–140 TDCs) across nearly all measures in the battery (significant). These tests are reliable (split half reliabilities >0.75) and have large normative (by gender and IQ) datasets from ages 6 to 18 (see Wolf et al., 2008). Based on this prior research, we chose the two LFI subtests which best discriminated the groups on face identity and face expression discrimination.

a. The Matching Identity Across Expression subtest evaluates a child's ability to recognize facial identities across changes in expression (happy, angry, sad, disgusted, and frightened). A target face is shown alone for 500 ms, followed by three probe faces of different identities presented simultaneously with the target face. Children must select the face that matches the target's identity ignoring the fact that the expression is different.
b. The Matchmaker Expression subtest assesses the child's ability to match emotional expressions across different identities. Five basic emotions (sad, angry, happy, frightened, and disgusted) were tested. A target face depicting a basic emotion in frontal profile was shown alone for 1000 ms and then remained on the screen as three probe faces of different identities conveying different expressions were presented. Children must select the face with the expression that matches the target.

#### Eye-tracking task

**Figure 1:**
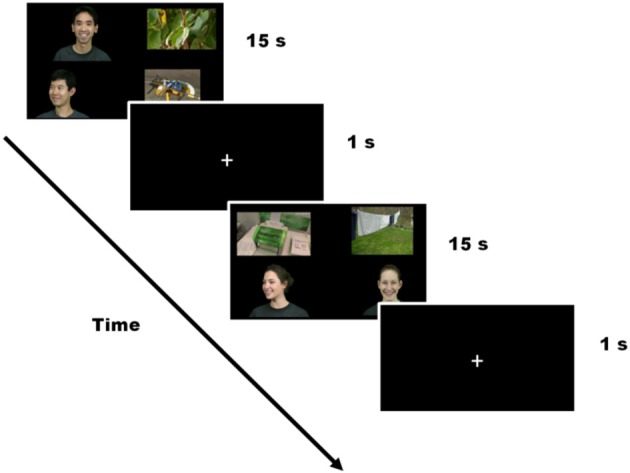
**Schematic representation of the experiment**.

Participants were calibrated at the beginning of the experiment using a standard five-point calibration procedure. The experiment included twelve 15-s trials consisting of four silent videos playing concurrently, one in each quadrant of the screen (pseudo-randomized location). In order to minimize the predictability of the display, a jitter was introduced so that the videos were not consistently placed right in the center of each quadrant. The distance in pixels from the center of the screen to the mid-point of each image did not differ between conditions [Face clips *M*_(*SD*)_ = 575(53)px; Object clips *M*_(*SD*)_ = 593(38)px; *t*_(46)_ = −1.69, *p* = 0.10]. The videos subtended approximately 20 degrees of visual angle horizontally and 14 degrees of visual angle vertically. The four videos shown on the screen in each trial consisted of (1) a face gazing directly at the camera, (2) a face averted from the camera (faces matched for sex), (3) a highly salient object, and (4) an object with lower salience. Face clips displayed emotions, which were the same within trial but different across trials. Twenty-four different faces were used (12 male, 12 female). Of the two faces in each trial, one faced the camera directly and was considered “high salience.” The other face was averted, and was considered “low salience.” Twenty-four different objects were included. Of the two objects in each trial, 12 were “high salience” including objects such as trains and airplanes (South et al., 2005). Twelve were “low salience” and included objects such as clothes and flowers. Each individual video clip lasted 3.75 s and was looped 4 times during the 15-s trial, so that children could look at each of the four clips and still get all of the visual information available in each clip. Trials were separated by a 1-s crosshair in the center of the screen (see Figure 1). Dynamic video stimuli fit a 2 × 2 design with Type (face/object) and Salience (high/low) as within-group factors.

### Procedure

At the beginning of each study visit, parents provide informed consent for their child; participant assent was obtained when feasible. Next the DAS-II and the ADOS were administered to the child while parents completed the ADI-R. After a lunch break, children completed the eyetracking task and the LFI tasks. Eye tracking took place in a quiet room containing a chair and a 30-inch computer screen on an adjustable table. A Tobii X120 gaze tracker recorded participants' looking patterns at a rate of 60 Hz from a seated distance of approximately 60 cm. Above the computer monitor, a webcam simultaneously recorded a video of the participant. Participants were informed that they would see a few short videos, and were asked to watch the screen.

All participants and parents received oral feedback at the time of the visit, as well as a written report, and compensation for time and travel. The Institutional Review Board at The Children's Hospital of Philadelphia approved all procedures related to this project.

### Preliminary analyses: *Let's Face It!* skills battery

Accuracy scores from the two *Let's Face It!* Identity and Expression subtests were averaged and examined for normality across 119 participants with and without ASD. Eight participants with the lowest scores (2 TDC, 6 ASD) and 1 with the highest score (TDC) were excluded as outliers. The remaining 110 participants had scores on the composite metric of face processing skills that met normality assumptions (Table 1; Shapiro-Wilk = 0.99, *p* = 0.26). Diagnostic group differences were found in the final sample such that TDC scored significantly higher (*M* = 82.70, *SD* = 7.78) than ASD (*M* = 78.83, *SD* = 7.29), *t*_(108)_ = 3.87, *p* = 0.008, Cohen's *d* = 0.51. This size of this difference is smaller than the one found by Tanaka and colleagues, for at least two reasons: First, children in the present study were included only if their Tobii sampling rate was above 80%, which already differentiates our sample from the original. However, a high sampling rate cutoff is required for accurate gaze data (which is a primary focus of our analyses). Second, eliminating outliers as necessary for the regression analyses we planned to run reduced variability and the size of the LFI group difference. Despite these limitations, the group difference in face processing as measured by the LFI was still of moderate effect size.

### Analyses

#### Eye tracking

Tobii software produces a variable called Total Fixation Duration, which is the sum total length of all fixations within a given AOI. It is often used as a measure of preference for looking at one stimulus type over another (Klin et al., 2002; Nakano et al., 2010; Rice et al., 2012). Given our hypothesis that the amount of time spent attending to a stimulus relates to the development of expertise in that stimulus type, we focused on Total Fixation Duration in our analyses. To control for individual variations in overall looking and to account for differences in AOI size, we calculated the Proportion of Total Fixation Duration by dividing the time spent looking at each AOI (high salience face, low salience face, high salience object, low salience object) by the total amount of time looking at all AOIs.

#### Statistics

Two types of analyses were performed. First, linear regressions were constructed to assess whether social attention predicts face processing skill and gaze to faces. Preliminary analyses revealed that age was significantly correlated with face processing skills (Pearson's *r* = 0.49, *p* < 0.001), so chronological age was entered in the first step of the regressions to control for its effect on face expertise. Second, a 2 (stimulus: face/object) × 2 (salience: high/low) × 2 (diagnostic group: ASD/TDC) repeated measures ANOVA explored whether gaze patterns differed for high- and low-salience stimuli, and whether looking patterns to faces and objects differed by diagnostic group. Stimulus type (face, object) and salience (high, low) were entered as within-subjects variables and diagnostic group (ASD, TDC) was entered as a between-subjects factor. Effect sizes (partial eta-squared, η^2^_p_, for F statistics and Cohen's *d* for *t*-tests) are reported together with *p*-values for significant main effects and interactions, and *post-hoc t*-tests are Bonferroni corrected to require a significance value of *p* < 0.01. A η^2^_p_ value above 0.01 is typically considered to reflect a small effect, a η^2^_p_ above 0.06 to reflect a medium effect, and a η^2^_p_ above 0.14 to reflect a large effect. Cohen's d values above 0.20, 0.50, and 0.80 are considered to reflect small, medium and large effects, respectively. The directionality of effects revealed by the omnibus ANOVA is determined using paired- and/or independent samples *t*-tests as appropriate.

### Test–retest reliability of the eye-tracking measure

Forty-two different participants (23 with ASD, 19 TDCs, all male, average age = 15.03 years, average IQ = 105.45) were recruited using the criteria described in the participant section above. These participants were asked to complete the eye tracking experiment at two time points separated by a 9-week interval (±1 week). An intraclass correlation coefficient (ICC) was computed using a two-factor mixed-effects consistency model (Farzin et al., 2011). An intraclass correlation >0.40 is considered good, and >0.75 is excellent. Test–retest reliability for the proportion of total fixation duration to faces at Time 1 and Time 2 was good to excellent (single measures ICC = 0.69, *p* < 0.001).

## Results

### Does gaze to faces predict face expertise?

**Figure 2:**
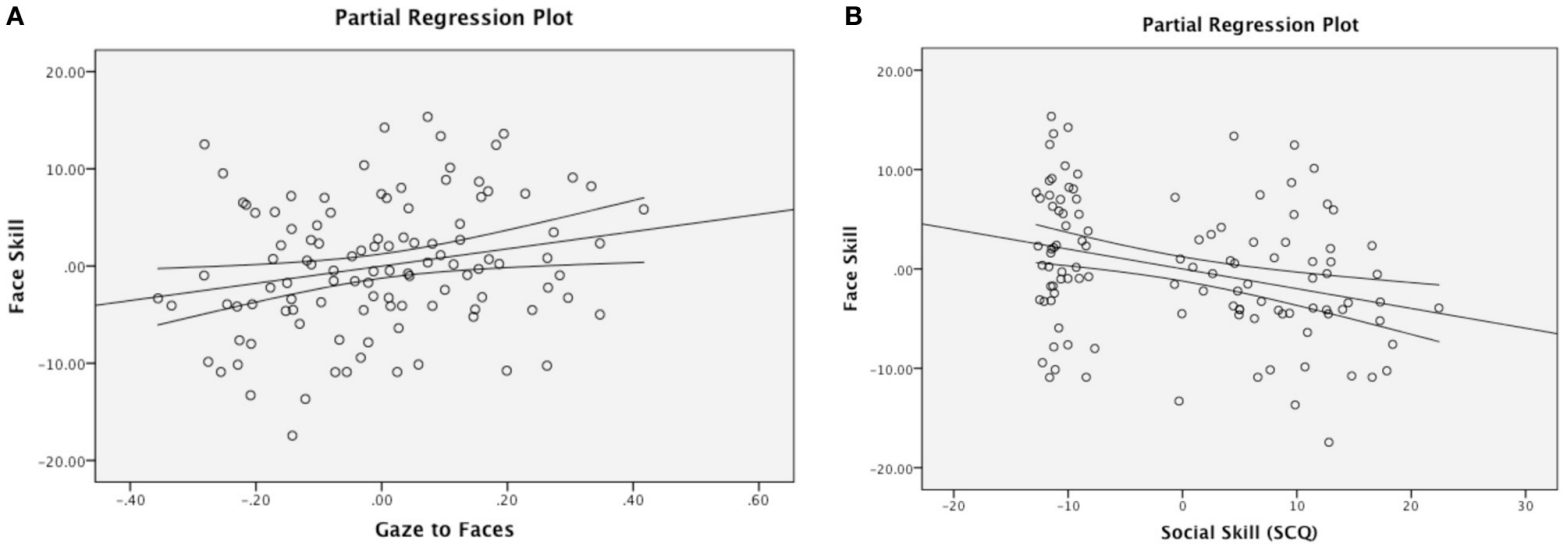
**Partial regression plots.** Gaze to faces predicting face skill **(A)** and social skill predicting face skill **(B)**, after controlling for the effect of chronological age. (**B**) Additionally illustrates a group difference in social skill.

The Social Motivation theory of autism argues that varying levels of social motivation modulate experience with faces over the course of development, and ultimately impact children's face processing skills. A two-step multiple regression analysis was therefore used to discern whether visual attention to faces predicts face perception skill in the combined sample of ASD and TDC participants. Age was entered in Step 1, as preliminary analyses suggested that face processing skills are positively correlated with chronological age (Pearson's *r* = 0.49, *p* < 0.001) and prior research suggests that face expertise continues to develop throughout childhood and adolescence (Carey et al., 1980; Thomas et al., 2007). Proportion of total fixation duration to faces was entered into the model in Step 2. Consistent with our hypothesis, attention to faces accounted for a significant amount of variance in face processing skills above and beyond the effect of age, Δ*F*_(1, 107)_ = 5.64, *p* = 0.02 (Table 3, Figure 2A).

**Table 3:** **Gaze predicts face processing skill—entire sample combined**.

**Variable**	**Beta**	***t*-value**	***p***	***R*^2^**	**Δ*R*^2^**
**STEP 1**					
Age	0.52	6.20	0.000	0.23	0.23
**STEP 2**					
Gaze to faces	0.27	2.38	0.019	0.27	0.04

Note: Beta is standardized.

Next, we tested whether scores on the SCQ (a measure that evaluates autistic symptomatology, including social communication skills) predicted total fixation duration to faces and face perceptual skills. While the SCQ not a measure of social motivation *per se*, these analyses may serve as a springboard for future targeted research using a scale designed specifically to assess motivation. A regression entering SCQ total score as a predictor of attention to faces returned a null result. Next, to test the relationship to face perception skill, we conducted a regression with age entered in Step 1 and SCQ entered in Step 2. Results revealed that the SCQ score accounts for a significant amount of variance in face processing skills after accounting for the effect of age, Δ*F*_(1, 107)_ = 23.92, *p* < 0.001 (Table 4, Figure 2B), with greater social impairment being associated with reduced face expertise.

**Table 4:** **Regression with SCQ score predicting face processing skill**.

**Variable**	**Beta**	***t*-value**	***p***	***R*^2^**	**Δ*R*^2^**
**STEP 1**					
Age	0.48	5.74	0.000	0.23	0.23
**STEP 2**					
SCQ score	−0.27	−3.41	0.000	0.31	0.08

Note: Beta is standardized.

To determine whether total fixation duration to faces differed by stimulus type, salience level, and diagnostic group, a 2 (Type: face/object) × 2 (Salience: high/low) × 2 (Diagnosis: ASD/TDC) repeated measures ANOVA was conducted. This analysis revealed a main effect of Type, *F*_(1, 108)_ = 61.63, *p* < 0.001, η^2^_p_ = 0.36, a main effect of Salience, *F*_(1, 108)_ = 131.07, *p* < 0.001, η^2^_p_ = 0.57, and an interaction between Type and Salience, *F*_(1, 108)_ = 44.17, *p* < 0.001, η^2^_p_ = 0.30. Contrary to our hypothesis, however, there was no effect of Diagnosis, either as a main effect or as an interaction with Type [*F*_(1, 108)_ = 0.13, *p* = 0.72, η^2^_p_ = 0.001], Salience [*F*_(1, 108)_ = 1.81, *p* = 0.18, η^2^_p_ = 0.02], or Type x Salience [*F*_(1, 108)_ = 0.27, *p* = 0.60, η^2^_p_ = 0.003]. *Post-hoc* tests revealed that all participants looked significantly more at objects (63%) than at faces (37%), *t*_(109)_ = −7.95, *p* < 0.001, and more at high salience stimuli (direct faces and high salience objects, 59%) than low salience stimuli (averted faces and low salience objects, 41%), *t*_(109)_ = 11.58, *p* < 0.001. Diagnostic group differences were not significant: participants with ASD looked at faces 36% of the time compared to 38% of the time in the TDC group, *t*_(108)_ = 0.37, *p* = 0.72, and at high salience stimuli (direct faces and high salience objects) 60% of the time compared to 50% in the TDC group, *t*_(108)_ = 1.35, *p* = 0.18. Interestingly, gaze to direct and averted faces was tightly correlated across groups (direct: 20%, averted: 17%, *r* = 0.73, *p* < 0.001) but gaze to high versus low salience objects was not (high salience: 39%, low salience: 24%, *r* = 0.10, *p* = 0.32). This suggests that high salience objects were much more riveting than low salience objects, and that all faces were attended to similarly whether they faced the observer or were averted.

We began our analyses with very strong a priori hypotheses about gaze in ASD versus TDC participants, based on a significant body of research (Klin et al., 2002; Nakano et al., 2010; Rice et al., 2012). Given that we purposefully calculated our eye tracking variables using Klin and colleagues' methods as a guide, the absence of diagnostic group differences was extremely surprising, and convinced us that the present data warranted a closer look. A number of strategies were used to probe the data and ensure that we did not miss a significant group difference in gaze. Our first follow-up analysis asked whether all children fixated on faces and objects equally quickly from the start of a trial or whether, perhaps, one group was slower to fixate on a certain stimulus type than the other. We hypothesized that the ASD group would fixate on objects more quickly than the TDC group, who would be faster to fixate on faces. As with Total Fixation Duration, however, there was no main effect of diagnosis, *F*_(1, 108)_ = 0.36, *p* = 0.55, and no interaction between diagnosis and Type, *F*_(1, 108)_ = 0.14, *p* = 0.71, or diagnosis and Salience, *F*_(1, 108)_ = 0.41, *p* = 0.52, or diagnosis, Type, and Salience, *F*_(1, 108)_ = 0.18, *p* = 0.67. Next we tested whether the ASD group might study faces and objects differently than the TDC group (e.g., by examining objects in greater detail than faces), which can be indexed by the number of times participants fixate within an AOI. Again, there was no interaction between diagnosis and Type, *F*_(1, 108)_ = 1.08, *p* = 0.30, or diagnosis and Salience, *F*_(1, 108)_ = 2.36, *p* = 0.13, or diagnosis, Type, and Salience, *F*_(1, 108)_ = 0.95, *p* = 0.33. We then tested the hypothesis that children with ASD would visit object AOIs more frequently than face AOIs, and that this pattern would be reversed in the TDC group. Results revealed no interaction between diagnosis and Type, *F*_(1, 108)_ = 2.20, *p* = 0.14, or diagnosis and Salience, *F*_(1, 108)_ = 1.55, *p* = 0.22, or diagnosis, Type, and Salience, *F*_(1, 108)_ = 0.10, *p* = 0.75. We further tested for differences in average visit duration. As with the other variables we explored, there was no interaction between diagnosis and Type, *F*_(1, 108)_ = 0.09, *p* = 0.76, or diagnosis and Salience, *F*_(1, 108)_ = 1.32, *p* = 0.25, or diagnosis, Type, and Salience, *F*_(1, 108)_ = 0.44, *p* = 0.51. Finally, although our sample is matched on chronological age and GCA at the group level, we re-ran the original RMANOVA on total fixation duration, including age and IQ as covariates in the model in addition to diagnosis as a fixed factor. The interaction between diagnosis and Type was still not significant, *F*_(1, 106)_ = 0.33, *p* = 0.57, nor was the interaction between diagnosis and Salience, *F*_(1, 106)_ = 1.59, *p* = 0.21, or diagnosis, Type, and Salience, *F*_(1, 106)_ = 0.21, *p* = 0.65.

#### First quartile

Long segments of gaze data may obscure meaningful eye movements that occur in the first few seconds of an experiment (Swingley et al., 1998). For this reason, we decided to isolate and examine the first 3.5-s loop of gaze data in each trial. A repeated measures ANOVA on proportion of total fixation duration in the first 3.5 s of each trial revealed no interaction between diagnosis and stimulus Type, *F*_(1, 108)_ = 1.39, *p* = 0.24, and no interaction between diagnosis and Salience, *F*_(1, 108)_ = 0.01, *p* = 0.92, or diagnosis, Type, and Salience, *F*_(1, 108)_ = 1.77, *p* = 0.19.

After exhausting the possibilities, we determined that our original finding, while surprising given the broader literature, was undeniably accurate. As discussed below, we speculate that the object movies in our paradigm may have been too appealing to reveal group differences that other paradigms with more subtle manipulations were able to document.

## Discussion

We aimed to answer three questions with this study: First, does visual attention to faces predict face expertise? Confirming our hypothesis, we found that increased gaze to faces relative to objects was a significant positive predictor of children's scores on the *Let's Face It!* Skills Battery. Although the effect is small, it represents an important first step toward understanding the relationship between social attention and one of our most fundamental areas of human expertise. Interestingly, even though the present eye tracking paradigm did not detect diagnostic group (categorical) differences, it was nonetheless sensitive to the dimensional relationship between gaze and face expertise. Future research will need to determine whether this relationship is stronger in different contexts, e.g., when using naturalistic interactive social scenes. More importantly, however, a longitudinal view must be taken. The current study took a cross-sectional approach and does not provide insight into how visual attention to faces contributes to growth in face expertise over the course of development.

Our second hypothesis, that social skill as measured by the SCQ would predict visual attention and face expertise, was partially confirmed. Although children's scores on the SCQ did not predict eye gaze, they did predict face expertise. One obvious limitation of this measure is that the SCQ is not specifically designed to gauge social motivation, which may explain the lack of correlation with visual attention. Future research using an instrument that measures social motivation more directly (such as the Pleasure Scale, Kazdin, 1989, used in ASD populations in Chevallier et al., 2012a,b) may clarify the relationship between motivation and gaze patterns.

Consistent with past work (Klin et al., 2002; Riby and Hancock, 2008; Rice et al., 2012), we asked whether children with ASD would look less at faces during a dynamic video presentation than TDCs. We hypothesized that this effect would be modulated by high versus low salient faces and objects. While there was a significant effect of movie salience, it did not interact with group; children in both diagnostic groups were very drawn to high-salience objects. In fact, participants were so attracted to the high salience stimulus set that there was little overall variance in gaze—most children looked at the high-salience objects the majority of the time. Had children been shown more engaging social stimuli (or less engaging non-social stimuli), diagnostic group differences might have emerged.

In conclusion, our study treated face processing skills as a dimension that spanned both children with ASD and TDC and found that amount of time spent looking at faces during eye tracking predicts face processing skill on an independent measure. This process-based analysis is consistent with a growing emphasis on using dimensional approaches in other areas of mental health research, as captured by the NIMH's new focus on research domain criteria (Insel et al., 2010; Sanislow et al., 2010). Exploring the diverse abilities of children with ASD with an eye toward incremental rather than categorical change has the potential to open new pathways to understanding the heterogeneity characteristic of this uniquely challenging, behaviorally defined disorder. Future research should study face processing longitudinally in large cohorts in order to better test the effect of differential attention to social objects on the development of face processing skills, using dynamic stimuli that span a wide range of salience.

### Conflict of interest statement

The authors declare that the research was conducted in the absence of any commercial or financial relationships that could be construed as a potential conflict of interest.
